# Patterns of PrEP and condom use among PrEP users in Belgium: a web-based longitudinal study

**DOI:** 10.1186/s12889-023-15786-6

**Published:** 2023-05-26

**Authors:** Anke Rotsaert, Tom Smekens, Bea Vuylsteke, Maarten Schim van der Loeff, Bernadette Hensen, Christiana Nöstlinger, Edwin Wouters, Jef Vanhamel, Gert Scheerder, Thijs Reyniers

**Affiliations:** 1 Department of Public Health, Institute of Tropical Medicine, Nationalestraat 155, Antwerp, 2000 Belgium; 2 Department of Infectious Diseases, Public Health Service of Amsterdam, Amsterdam, The Netherlands; 3 Department of Internal Medicine, Amsterdam UMC location University of Amsterdam, Meibergdreef 9, Amsterdam, The Netherlands; 4 Amsterdam Institute for Infection and Immunity (AII), Amsterdam, the Netherlands; 5 Amsterdam Public Health Research Institute (APH), Amsterdam, the Netherlands; 6 Department of Sociology, University of Antwerp, Antwerp, Belgium

**Keywords:** PrEP use, Longitudinal, Condom use, MSM, Belgium, HIV prevention

## Abstract

**Background:**

Tailoring pre-exposure prophylaxis (PrEP) service delivery is key to scaling-up PrEP uptake. Optimal implementation of tailored services requires, among other things, insights into patterns of PrEP use, sexual behaviours and condom use over time.

**Methods:**

Between September 2020 and January 2022, we conducted a web-based, longitudinal study among PrEP users in Belgium. In three questionnaire rounds every six-months, we assessed PrEP and condom use, and sex with steady, casual and anonymous partners in the preceding three months. Based on the patterns of PrEP use in the preceding three months, we identified distinct PrEP use categories. We investigated differences in baseline socio-demographics and sexual behaviours by PrEP use category using Fisher’s exact and one-way ANOVA tests. Patterns in PrEP and condom use over time were examined using descriptive analyses and visualised in alluvial diagrams.

**Results:**

In total, 326 participants completed the baseline questionnaire, and 173 completed all three questionnaires. We identified five distinct PrEP use categories: daily (≥ 90 pills), almost daily (75–89 pills), long period (> 7 consecutive days and < 75 pills) with or without additional short period use, short period (1–7 consecutive days and < 75 pills) and no PrEP use (0 pills). During the study, percentages of individuals in each PrEP use category varied, but did not change significantly over time. At baseline, daily and almost daily users were more likely to report five or more casual sex partners, ten or more anonymous sex partners and anal sex on a weekly basis with casual or anonymous partners compared to those using PrEP for long or short periods. Up to 12.6% (n = 16/127) of participants reporting anal sex with casual or anonymous partners, indicated always using condoms and PrEP with these partners. One in three (n = 23/69) participants who reported anal sex with steady partners had condomless anal sex and did not use PrEP with these partners; with casual or anonymous partners less than 3% reported this.

**Conclusions:**

Our findings show that there is little variation in PrEP use over time and that PrEP use was associated with sexual behaviours, which could be taken into account when designing tailored PrEP care.

**Supplementary Information:**

The online version contains supplementary material available at 10.1186/s12889-023-15786-6.

## Introduction

To achieve the UNAIDS target of fewer than 370 000 annual new HIV infections globally by 2025, efficacious HIV prevention tools, including oral pre-exposure prophylaxis (PrEP), need to be scaled up [1, 2]. Achieving equitable scale-up requires innovative delivery approaches that are differentiated, tailored, and adopt a person-centred focus [3]. Enabling tailored care, which is adapted to the needs and preferences of its users, requires in addition to other information, insights into patterns of PrEP use.

PrEP demonstration projects and cohort studies have shown that PrEP users can safely switch between daily and on-demand regimens or temporarily discontinue PrEP [4–6]. In response to these findings, the World Health Organization (WHO) recommends that cisgender men, and trans and gender diverse people assigned male at birth, who are not taking exogenous estradiol-based hormones, are eligible for either daily or on-demand PrEP [3]. In practice, PrEP users often adapt their PrEP use according to changes in sexual behaviours and perceived prevention needs [7]. As such, distinguishing between daily and on-demand use may be less straightforward. Exploring patterns of PrEP use and associated factors may help inform how PrEP services can be adapted for different types of users with differing needs.

Various PrEP implementation and open-label studies have demonstrated increased number of sexual partners [4, 5], reduced condom use [4, 5, 8], and increased sexual well-being [9, 10] among PrEP users. Although some studies have shown that PrEP users continue to use condoms, either consistently or in certain settings or with certain types of sexual partners, as a viable option to prevent HIV and other sexually transmitted infections (STIs) [7, 11], there may be an evolving shift in PrEP users’ social norms regarding condom use and thus in the notion of ‘safe sex’ [11–13]. With the expansion of HIV prevention options, ‘safe sex’ no longer only implies condom use [13]. Ineffective PrEP use combined with reduced condom use poses an HIV acquisition risk for MSM [14]. Therefore, to fully understand the implications of PrEP use patterns on potential HIV risk, it is important to assess sexual behaviour, in particular condom use and how PrEP and condoms are combined over time [15]. This will help to design appropriate counselling strategies and prevention interventions.

MSM are a priority population for PrEP in Europe, including Belgium [16], where almost 5300 individuals have started PrEP since its roll-out in 2017 [17]. Despite evidence that 58% of individuals starting PrEP opted for on-demand PrEP in 2021 [17], there are few insights into actual patterns of PrEP and condom use over time among PrEP users in Belgium [6]. The objectives of this study were to describe patterns of PrEP use over time, to examine socio-demographic and sexual behaviour factors associated with PrEP use, and to describe PrEP and concurrent condom use by partner type over time among PrEP users in Belgium in order to inform PrEP programmes.

## Methods

### Study design

Between September 2020 and January 2022, we conducted a web-based, longitudinal study consisting of three rounds of questionnaires among PrEP users in Belgium.

### Data collection

We recruited participants through the social media platforms of MSM community organisations, HIV/STI clinics and through social and sexual networking apps. Eligibility criteria were being 16 years or older, having a self-reported HIV negative or unknown HIV serostatus, living in Belgium and having used PrEP in the six months prior to the baseline questionnaire. In Belgium, the minimum age to be eligible for PrEP and to be entitled for reimbursement is 16. The minimum age at which an individual is legally considered old enough to consent to participation in sexual activities is also 16 years. With regard to participating in online research, the minimum age of consent for processing personal data is 13. After the baseline questionnaire, participants were invited, via email, to complete two follow-up questionnaires (FU1 and FU2) at six-month intervals. Up to two reminders were sent in case of non-response. Questionnaires were available in Dutch, French and English.

### Measures and definitions

The baseline questionnaire included modules on: socio-demographics (age, nationality, education, occupational and financial status, social health insurance, sex assigned at birth) and sexuality. We measured sexual health based on the WHO framework of sexual health indicators, including sexual satisfaction, safety and autonomy [18] with a scale of four items measuring sexual satisfaction, sexual safety and sexual autonomy (e.g. ‘I’m happy with my sex life’; ‘the sex I have is always as safe as I want it to be’). These four items were derived from a previous study, using four 5-point Likert items ranging from ‘strongly disagree’ (1) to ‘strongly agree’ (5, 9). (Additional file 1) The mean of these four items was used to estimate the sexual health score; higher means indicate better sexual health. Furthermore, we assessed the propensity to attain optimal levels of sexual excitement and to engage in novel sexual experiences using the 10-item sexual sensation-seeking scale (SSSS) ranging from ‘not at all like me’ (1) to ‘very much like me’ (4). (Additional file 2) The mean of the ten items was the sexual sensation score; higher mean values indicates scoring higher on the sexual sensation-seeking scale [19].

At baseline, we assessed time since PrEP start (options provided: <6 months, 6–12 months, 12–24 months, > 24 months ago) and PrEP use in the preceding three months by asking about type of PrEP regimen chosen (options provided: daily, non-daily, no PrEP). For more nuanced data on PrEP use, we adapted the PrEP use question in the questionnaires for FU1 and FU2. Here, we asked about the number of PrEP pills taken in the preceding three months (options provided: 0, 1–7, 8–29, 30–74, 75–90). Participants who indicated daily PrEP use at baseline or to have taken between 75 and 90 PrEP pills at FU1 or FU2, completed a follow-up question on the number of days when they did not use PrEP in the preceding three months. Participants who indicated non-daily PrEP use at baseline or to have taken between one and 74 PrEP pills in FU1 or FU2, completed follow-up questions on length and frequencies of periods of use, i.e. whether PrEP was used for more than seven consecutive days or for a maximum of seven consecutive days.

Based on participants’ responses, we constructed the following five mutually exclusive PrEP use categories per three months: (1) daily (at least 90 pills taken), (2) almost daily (75–89 pills taken), (3) long period (more than 7 consecutive days of use and < 75 pills taken overall) with or without additional short period use, (4) short periods only (1 to 7 consecutive days of use and < 75 pills taken overall), and (5) no PrEP use (zero pill intake). Inconsistent answers were coded as missing.

In all three questionnaires, we asked about sexual behaviours with steady, casual and anonymous sex partners in the preceding three months. Having a steady partner was defined as “not being single and considering yourself to have a serious relationship with someone (e.g. husband, wife), whereby length of the relationship did not matter”. A casual sex partner was described as a person with whom “you have regular sex but not a steady relationship, but who is not anonymous”. An anonymous sex partner was defined as a person who “you do not know or you just got to know”. For each type of partner we asked about the number of partners (we provided predefined options per partner type), frequency of anal sex (daily, weekly, monthly, less than monthly), frequency of condom use (never, sometimes, always) and PrEP use (never, sometimes, always) during anal sex. At each study round, participants were asked whether they had been diagnosed with an STI in the preceding six months (options provided: yes, no).

For each questionnaire and each partner type, we combined the responses on frequency of condom use and PrEP use during anal sex into nine condom and PrEP use categories. For example, participants reporting never using condoms, but always using PrEP, during anal sex with casual partners, were combined into a variable of ‘never using condoms, always using PrEP’ with casual partners.

### Data analysis

We compared baseline socio-demographics and sexual behaviours between three types of PrEP users: (1) daily users, which combines daily and almost daily users, (2) long period users with or without additional short period use, referred to as long period users, (3) short period users only, using Fisher’s exact and one-way ANOVA tests.

We restricted subsequent analyses to participants who completed all three questionnaires rounds. To explore potential attrition bias, we compared baseline characteristics of participants who completed all three study rounds to those who did not, using Pearson’s Chi-square, Fisher’s exact and Wilcoxon rank sum tests, as appropriate. We examined patterns of PrEP use at each study round using the five defined PrEP use categories and visualised patterns of PrEP use over the study period in alluvial diagrams using the ‘ggalluvial’ R package [20]. Across the three study rounds, we assessed whether the percentage of individuals in each PrEP use category changed over time when compared to all other PrEP use categories combined, using a Chi-square test with Rao and Scott adjustment for repeated measures [21]. Similarly, we examined patterns in condom and PrEP use combinations per partner type at each study round and visualised these patterns using alluvial diagrams. For this analysis, we excluded participants who either never reported having a particular partner or never having had anal sex with such a partner in all the three rounds. We assessed whether the percentage of individuals in each condom and PrEP use combination category changed over time when compared to all other condom and PrEP use combination categories combined, using a Chi-square test with Rao and Scott adjustment for the repeated measures [21]. We used R statistical software version 4.0.2 for all analyses [22].

### Ethics approval

Potential participants provided consent by agreeing to participate, after having been informed about the study and its procedures. The study received ethical approval through the Institutional Review Board of the Institute of Tropical Medicine, Antwerp (IRB 1380/20 and IRB 1352/20).

## Results

### Study population

Among the 326 participants who completed the baseline questionnaire, 208 (63.8%) completed the FU1, and 186 (57.1%) FU2. About one in five (21.5%, n = 70) baseline participants did not consent or did not provide their contact details for follow-up. Approximately half (53.1%, n = 173) of the baseline participants completed all three study rounds. Among baseline participants, almost all were male (99.1%, n = 323) and sexually attracted to men (98.8%, n = 322). Their median age was 42 years (IQR 34–50; Table 1). Most were born in Belgium (85.6%, n = 279), had a higher education (81.6%, n = 266) and were employed (82.6%, n = 269). Almost half reported to be “living comfortably” on their current income (47.2%, n = 154) and that they had initiated PrEP more than 24 months ago (46.0%, n = 150). About one in three (30.1%, n = 98) reported an STI diagnosis in the preceding six months.

**Table 1:** Sociodemographic and sexual behaviour characteristics, reported sexually transmitted infections and PrEP use at baseline of all participants at baseline (N = 326), participants completing all study rounds (N = 173) and those who did not (N = 153); study on PrEP users’ patterns of PrEP use, Belgium, 2020–2022

	All participants at baseline N = 326	Participants who completed all study rounds N = 173	Participants who did not complete all study rounds N = 153		
	n (%)	n (%)	n (%)		P-value^£^
**Sociodemographic characteristics**					
Age in years, median [IQR]	42 [34–50]	44 [36–52]	38 [32–48]		**< 0.001**
Born in Belgium	279 (85.6)	151 (87.3)	128 (83.7)		0.440
Higher education^1^	266 (81.6)	136 (78.6)	130 (85.0)		0.182
Occupational status^2^					0.779
Employed	269 (82.5)	142 (82.1)	127 (83.0)		
Unemployed	52 (16.0)	29 (16.8)	23 (15.0)		
Other	5 (1.5)	2 (1.1)	3 (2.0)		
Financial status					0.208
Living really comfortably on present income	65 (19.9)	31 (17.9)	34 (22.2)		
Living comfortably on present income	154 (47.2)	84 (48.6)	70 (45.8)		
Neither comfortable nor struggling on present income	75 (23.0)	45 (26.0)	30 (19.6)		
Struggling or really struggling on present income	26 (8.0)	12 (6.9)	14 (9.2)		
Preferred not to say	6 (1.8)	1 (0.6)	5 (3.3)		
Having social health insurance	320 (98.2)	169 (97.7)	151 (98.7)		0.688
**Sex and sexuality**					
Male sex at birth	323 (99.1)	171 (98.8)	152 (99.3)		1
Sexually attracted to men	322 (98.8)	171 (98.8)	151 (98.7)		1
Sexual health score, median [IQR]	3.7 [3.3–4.3]	3.8 [3.3–4.3]	3.8 [3.3–4.3]		0.539
Sexual sensation seeking scale score, median [IQR]	2.9 [2.5–3.2]	2.9 [2.5–3.2]	2.8 [2.5–3.2]		0.895
**PrEP use**					
First started taking PrEP					0.125
Less than 6 months ago	21 (6.4)	6 (3.5)	15 (9.8)		
6–12 months ago	49 (15.0)	25 (14.4)	24 (15.7)		
12–24 months ago	106 (32.5)	58 (33.5)	48 (31.4)		
More than 24 months ago	150 (46.0)	84 (48.6)	66 (43.1)		
PrEP use in the preceding 3 months					**0.032**
Daily/almost daily	142 (43.6)	77 (44.5)	65 (42.5)		
Long period use with or without additional short period use	64 (19.6)	26 (15.0)	38 (24.8)		
Short period use only	83 (25.5)	54 (31.2)	29 (19.0)		
No PrEP	13 (4.0)	5 (2.9)	8 (5.2)		
Missing	24 (7.4)	11 (6.4)	13 (8.5)		
**Sexually transmitted infections**					
Reported having had an STI in the preceding 6 months	98 (30.1)	50 (28.9)	48 (31.4)		0.716
**Sexual behaviour in the preceding 3 months**					
**STEADY PARTNERS**					
Number of steady partners					0.906
0	162 (49.7)	87 (50.3)	75 (49.0)		
1 or more	164 (50.3)	86 (49.7)	78 (51.0)		
Anal sex with steady partners*					0.735
No	43 (26.2)	24 (27.9)	19 (24.4)		
Yes	121 (73.8)	62 (72.1))	59 (75.6)		
Condom use during anal sex with steady partners**					0.692
Always	4 (3.3)	3 (4.8)	1 (1.7)		
Sometimes	11 (9.1)	5 (8.1)	6 (10.2)		
Never	106 (87.6)	54 (87.1)	52 (88.1)		
PrEP use during anal sex with steady partners**					0.691
Always	66 (54.5)	35 (56.5)	31 (52.5)		
Sometimes	12 (9.9)	7 (11.3)	5 (8.5)		
Never	43 (35.5)	20 (32.3)	23 (39.0)		
**CASUAL SEX PARTNERS**					
Number of casual partners					0.076
0	38 (11.7)	16 (9.2)	22 (14.4)		
1	24 (7.4)	17 (9.8)	7 (4.6)		
2–4	101 (31.0)	48 (27.7)	53 (34.6)		
5 or more	163 (50.0)	92 (53.2)	71 (46.4)		
Frequency of anal sex with casual partners*					0.292
No anal sex	27 (9.4)	14 (8.9)	13 (9.9)		
Less than monthly	46 (16.0)	29 (18.5)	17 (13.0)		
Monthly	111 (38.5)	58 (36.9)	53 (40.5)		
Weekly	101 (35.1)	56 (35.7)	45 (34.4)		
Daily	3 (1.0)	0 (0.0)	3 (2.3)		
Condom use during anal sex with casual partners**					0.203
Always	27 (10.3)	12 (8.4)	15 (12.7)		
Sometimes	113 (43.3)	58 (40.6)	55 (46.6)		
Never	121 (46.4)	73 (51.0)	48 (46.8)		
PrEP use during anal sex with casual partners**					**0.013**
Always	237 (90.8)	136 (95.1)	101 (85.6)		
Sometimes	20 (7.7)	5 (3.5)	15 (12.7)		
Never	4 (1.5)	2 (1.4)	2 (1.7)		
**ANONYMOUS SEX PARTNERS**					
Number of anonymous partners					0.612
0	75 (23.0)	34 (19.7)	41 (26.8)		
1	23 (7.1)	14 (8.1)	9 (5.9)		
2–5	122 (37.4)	66 (38.2)	56 (36.6)		
6–9	25 (7.7)	14 (8.1)	11 (7.2)		
10 or more	81 (24.8)	45 (26.0)	36 (23.5)		
Frequency of anal sex with anonymous partners*					0.135
No anal sex	27 (10.8)	11 (8.0)	15 (13.4)		
Less than monthly	56 (22.3)	35 (25.4)	21 (18.7)		
Monthly	92 (36.7)	55 (39.9)	37 (32.0)		
Weekly	74 (29.5)	37 (26.8)	37 (33.0)		
Daily	2 (0.8)	0 (0.0)	2 (1.8)		
Condom use during anal sex with anonymous partners**					0.176
Always	36 (16.1)	16 (12.6)	20 (20.6)		
Sometimes	90 (40.2)	50 (39.4)	40 (41.2)		
Never	98 (43.8)	61 (48.0)	37 (38.1)		
PrEP use during anal sex with anonymous partners**					**< 0.001**
Always	205 (91.5)	124 (97.6)	81 (83.5)		
Sometimes	15 (6.7)	3 (2.4)	12 (12.4)		
Never	4 (1.8)	0 (0.0)	4 (4.1)		

*IQR: interquartile range, PrEP: Pre-Exposure Prophylaxis, STIs: sexually transmitted infections, £: using Pearson’s Chi-square, Fisher’s exact or Wilcoxon rank sum tests. 1: higher education means college or university and includes higher education long type (i.e. more than 3 years) and short type (i.e. 3 years or less), 2: occupational status: ‘unemployed’ includes long-term sick/leave/medically retired, technical unemployed, retired, student, and unemployed. ‘employed’ includes employed full-time, employed part-time and self-employed. Values in bold indicate significant p-values < 0.05*

* Among those with respectively steady, casual or anonymous sex partners

** Among those who report anal sex with respectively steady, casual or anonymous sex partner

Compared to participants who did not complete all study rounds, participants who completed all three rounds were more likely to be older (median age 44 years vs. 38 years) (p < 0.001), to use PrEP more for short periods only (31.2% vs. 19.0%) (p = 0.032) and to report consistent PrEP use for anal sex with casual (95.1% vs. 85.6%) (p = 0.013) and anonymous sex partners (97.6% vs. 83.5%) (p < 0.001). Participants who completed the three study rounds had more casual sex partners, but there was no evidence that this difference was statistically significant. (Table 1)

### Associations between PrEP use categories at baseline and socio-demographics and sexual behaviours

At baseline, 142 (43.6%) participants reported taking PrEP daily or almost daily, 64 (19.6%) reported using PrEP for long periods and 83 (25.5%) used PrEP for short periods only. (Table 2) Thirteen participants reported no PrEP use in the preceding three months and the responses of 24 participants were inconsistent. Daily PrEP users were more likely to score higher on the sexual health scale (mean = 3.9) than long (mean = 3.6) and short period users (mean 3.7) (p = 0.002).

**Table 2:** Sociodemographics, reported sexually transmitted infections and sexual behaviours by PrEP use category in the preceding three months (i.e. daily (N = 142), long period (N = 64), and short period (N = 83) use) at baseline and associations between these factors and PrEP use category; study on PrEP users’ patterns of PrEP use, Belgium, 2020–2022

	Daily/almost daily N = 142	Long period use with or without additional short period use N = 64	Short period use only N = 83	p-value^£^
	n (%)	n (%)	n (%)	
**Sociodemographic baseline characteristics**				
Age in years, mean (SD)	41.4 (9.4)	41.0 (9.4)	43.8 (10.7)	0.143
Born in Belgium	117 (82.4)	56 (87.5)	75 (90.4)	0.247
Higher education^1^	111 (78.2)	52 (81.3)	70 (84.3)	0.531
Occupational status^2^				0.365
Employed	122 (85.9)	54 (84.4)	66 (79.5)	
Unemployed	19 (13.4)	10 (15.6)	14 (16.9)	
Other	1 (0.7)	0 (0.0)	3 (3.6)	
Financial status				0.072
Living really comfortably on present income	30 (21.1)	14 (21.9)	12 (14.5)	
Living comfortably on present income	68 (47.9)	29 (45.3)	39 (47.0)	
Neither comfortable nor struggling on present income	25 (17.6)	18 (28.1)	26 (31.3)	
Struggling or really struggling on present income	18 (12.7)	2 (3.1)	4 (4.8)	
I prefer not to say	1 (0.7)	1 (1.6)	2 (2.4)	
Having social health insurance	139 (97.9)	62 (96.9)	83 (100.0)	0.304
**Sex and sexuality**				
Male sex at birth	141 (99.3)	63 (98.4)	83 (100.0)	0.461
Sexually attracted to men	141 (99.3)	62 (96.9)	83 (100.0)	0.204
Sexual health score (scale 1–5), mean (SD)	3.9 (0.7)	3.6 (0.6)	3.7 (0.7)	**0.002**
Sexual sensation seeking scale score (scale 1–4), mean (SD)	2.9 (0.5)	2.8 (0.4)	2.8 (0.5)	0.174
**Time since PrEP start**				
First started taking PrEP				**0.046**
Less than 6 months ago	13 (9.2)	3 (4.7)	5 (6.0)	
6–12 months ago	19 (13.4)	9 (14.1)	14 (16.9)	
12–24 months ago	37 (26.1)	32 (50.0)	27 (32.5)	
More than 24 months ago	73 (51.4)	20 (31.3)	37 (44.6)	
**Sexually transmitted infections**				
Reported having had an STI in the preceding 6 months	50 (35.2)	20 (31.3)	19 (22.9)	0.157
**Sexual behaviour in the preceding 3 months**				
**STEADY PARTNER**				
Number of steady partners				0.879
0	72 (50.7)	30 (46.9)	40 (48.2)	
1 or more	70 (49.3)	34 (53.1)	43 (51.8)	
Anal sex with steady partners*				0.162
No	15 (21.4)	15 (44.1)	9 (20.9)	
Yes	55 (78.6)	19 (55.9)	34 (79.1)	
Condom use during anal sex with steady partners**				0.234
Always	2 (3.6)	0 (0.0)	1 (2.9)	
Sometimes	4 (7.3)	4 (21.1)	3 (8.8)	
Never	49 (89.1)	15 (78.9)	30 (88.2)	
PrEP use during anal sex with steady partners**				**< 0.001**
Always	47 (85.5)	4 (21.1)	9 (26.5)	
Sometimes	2 (3.6)	5 (26.3)	4 (11.8)	
Never	6 (10.9)	10 (52.6)	21 (61.8)	
**CASUAL SEX PARTNER**				
Number of casual partners				**< 0.001**
0	6 (4.2)	7 (10.9)	11 (13.3)	
1	7 (4.9)	5 (7.8)	11 (13.3)	
2–4	31 (21.8)	23 (35.9)	37 (44.6)	
5 or more	98 (69.0)	29 (45.3)	24 (28.9)	
Frequency of anal sex with casual partners*				**< 0.001**
No anal sex	10 (7.4)	7 (10.9)	7 (9.7)	
Less than monthly	14 (10.3)	5 (8.8)	21 (29.2)	
Monthly	42 (30.9)	30 (52.6)	31 (43.1)	
Weekly	68 (50.0)	15 (26.3)	13 (18.1)	
Daily	2 (1.5)	0 (0.0)	0 (0.0)	
Condom use during anal sex with casual partners**				0.160
Always	12 (9.5)	4 (8.0)	8 (12.3)	
Sometimes	58 (46.0)	25 (50.0)	22 (33.8)	
Never	56 (44.4)	21 (42.0)	35 (53.8)	
PrEP use during anal sex with casual partners**				**0.005**
Always	121 (96.0)	45 (90.0)	55 (84.6)	
Sometimes	3 (2.4)	5 (10.0)	10 (15.4)	
Never	2 (1.6)	0 (0.0)	0 (0.0)	
**ANONYMOUS SEX PARTNER**				
Number of anonymous partners				**< 0.001**
0	16 (11.3)	11 (17.2)	33 (39.8)	
1	7 (4.9)	3 (4.7)	10 (12.0)	
2–5	51 (35.9)	30 (46.9)	28 (33.7)	
6–9	13 (9.2)	7 (10.9)	4 (4.8)	
10 or more	55 (38.7)	13 (20.3)	8 (9.6)	
Frequency of anal sex with anonymous partners*				**< 0.001**
No anal sex	10 (7.9)	7 (13.2)	4 (8.0)	
Less than monthly	23 (18.3)	9 (17.0)	18 (36.0)	
Monthly	38 (30.2)	28 (52.8)	22 (44.0)	
Weekly	54 (42.9)	9 (17.0)	6 (12.0)	
Daily	1 (0.8)	0 (0.0)	0 (0.0)	
Condom use during anal sex with anonymous partners**				**< 0.001**
Always	15 (12.9)	7 (15.2)	10 (21.7)	
Sometimes	51 (44.0)	22 (47.8)	12 (26.1)	
Never	50 (43.1)	17 (37.0)	24 (52.2)	
PrEP use during anal sex with anonymous partner**				**< 0.001**
Always	112 (96.6)	39 (84.8)	40 (87.0)	
Sometimes	3 (2.6)	6 (13.0)	5 (10.9)	
Never	1 (0.9)	1 (2.2)	1 (2.2)	

*SD: standard deviation, PrEP: Pre-Exposure Prophylaxis, STIs: sexually transmitted infections, £ using Fisher’s exact or one-way ANOVA tests. 1: higher education means college or university and includes higher education long type (i.e. more than 3 years) and short type (i.e. 3 years or less), 2: occupational status: ‘unemployed’ includes long-term sick/leave/medically retired, technical unemployed, retired, student, and unemployed. ‘employed’ includes employed full-time, employed part-time and self-employed., values in bold indicate significant p-values < 0.05*

* Among those with respectively steady, casual or anonymous sex partners / ** Among those who report anal sex with respectively steady, casual or anonymous sex partners

About half of daily (51.4%, n = 73) and short period users (44.6%, n = 37) had started taking PrEP more than two years ago, versus 31.3% (n = 20) of long period users (p = 0.046). Daily users were more likely to report five or more casual sex partners (69.0%, n = 98 vs. 45.3%, n = 29; 28.9% n = 24, respectively), and a higher frequency of anal sex with casual partners compared to long and short period users in the preceding three months (p < 0.001). Short period users were less likely to report anonymous sex partners and frequent anal sex with anonymous partners compared to daily or long period users (p < 0.001). Although less likely to report anonymous partners, short period users were more likely to report never using condoms during anal sex with anonymous sex partners (52.2%, n = 24) compared to daily (43.1%, n = 50) or long period users (37.0%, n = 17) (p < 0.001).

### Patterns of PrEP use over time

**Fig. 1:**
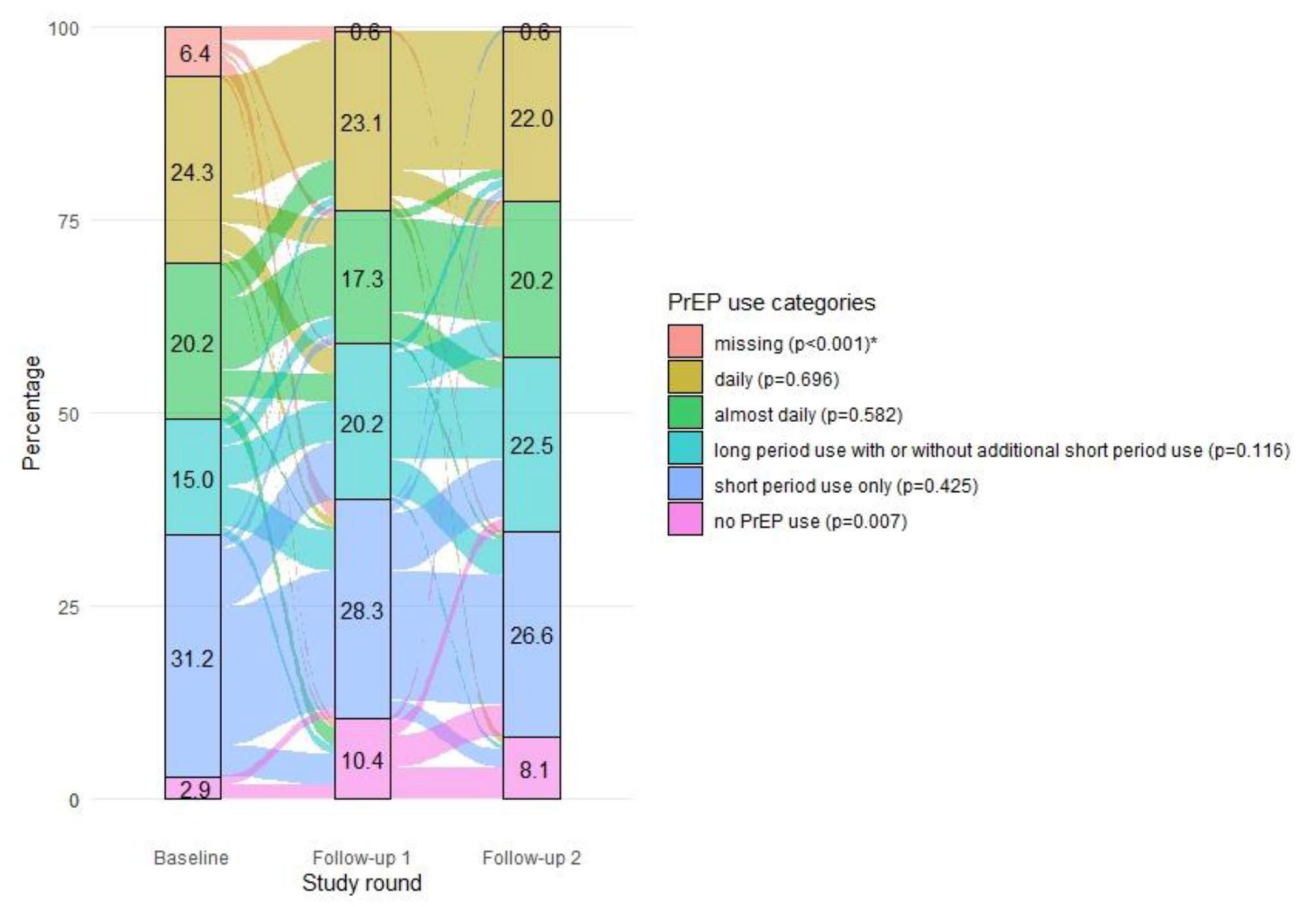
Alluvial diagram of PrEP use over time, N = 173 *PrEP use in the preceding three months: daily: ≥ 90 pills; almost daily: 75–89 pills; long period use with or without additional short period use: using PrEP in episodes of more than 7 days in a row and less than 75 pills in total, with or without additional short period use; short period use only: taking PrEP in episodes of 1 up to 7 consecutive days and less than 75 pills; None: zero pills* * The p-value between brackets represents the p-value of the Chi-squared test with Rao and Scott adjustment. This test assessed whether the proportion of each PrEP use category differed significantly over time when compared to all other PrEP use categories combined

Figure 1 shows participants’ transitions between PrEP use categories over the study period. At baseline, 42 (24.3%) participants reported using PrEP daily, 35 (20.2%) almost daily, 26 (15.0%) for long periods (with or without additional short period use) and 54 (31.2%) for short periods only. These percentages did not change significantly over time. Among those who switched PrEP categories, transitions most often occurred to an adjacent category. For example, between FU1 and FU2, eight of the 19 participants who transitioned from long period use did so to short period use. The number of participants reporting no PrEP use in the preceding three months changed significantly over time from, from five at baseline to 18 at FU1 to 14 at FU2 (p = 0.007). Participants who reported no PrEP use in the preceding three months mainly reported short period use in previous rounds (Baseline: n = 7, FU1: n = 4).

### Condom and PrEP use by partner type over time

**Fig. 2:**
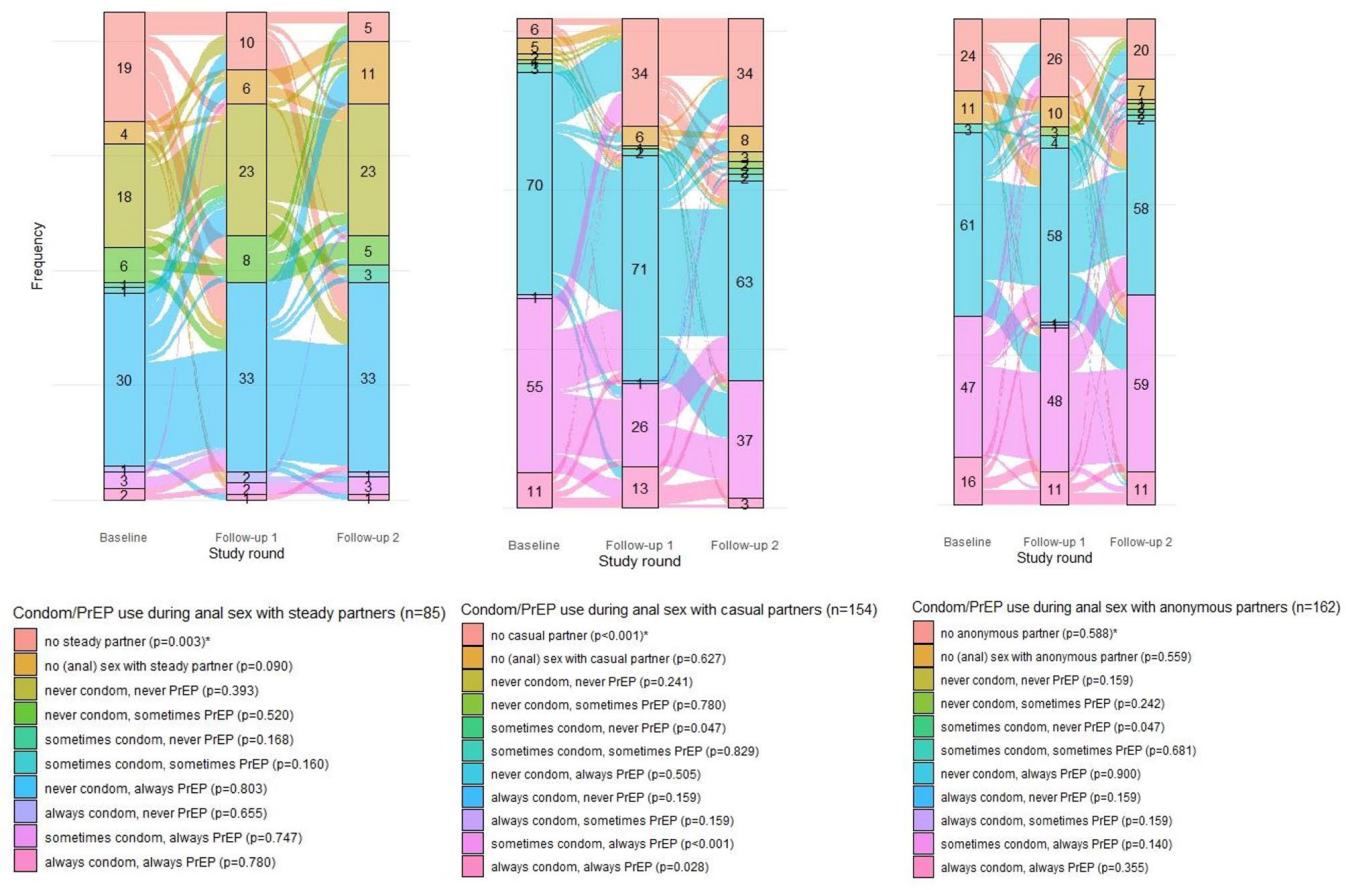
Alluvial diagrams of condom and PrEP use during anal sex with steady (n = 85)^£^, casual (n = 154)^£^, and anonymous partners (n = 162)^£^ over time * The p-value between brackets represents the p-value of the chi-square test with Rao and Scott adjustment. This test assessed whether the proportion of each condom and PrEP use combination category differed significantly over time when compared to all other condom and PrEP use combination categories combined *£ 173 participants completed all three study rounds. For the analysis of PrEP and condom use with one particular partner type we excluded participants who either never reported having such partner or reported never having had anal sex with such a partner during the three study rounds. Therefore numbers vary by partner type. None of the participants reported always using condoms and sometimes using PrEP during anal sex with steady partners, therefore, this category is not reported in the alluvial diagram about condom and PrEP use during anal sex with steady partners*

Figure 2 shows participants’ transitions between combined condom and PrEP use categories by partner type across the study. Among all participants who completed the three questionnaires, 85 (49.1%) reported anal sex with a steady partner in the preceding three months in one or more questionnaires. Among the 62 participants who reported anal sex with a steady partner at baseline half (48.4%, n = 30/62) reported never using condoms and always using PrEP, almost one-third (29.0%, n = 18/62) reported never using condoms or PrEP and 9.7% (n = 6/62) reported never using condoms and sometimes using PrEP. While these percentages remained relatively consistent over time, the percentage of participants reporting no steady partner decreased from 22.4% (n = 19) to 5.9% (n = 5) between baseline and FU2 (p = 0.003).

During the study period, 154 (89.0%) participants reported anal sex with a casual partner in one or more questionnaires. Between half and almost two-thirds of these individuals reported never using condoms and always using PrEP (Baseline: 49.0%, n = 70/143; FU1: 62.3%, n = 71/114; FU2: 56.3%, n = 63/112), with between one-fifth and one-third reporting sometimes using condoms and always using PrEP (Baseline: 38.5%, n = 55/143; FU1: 22.8%, n = 26/114; FU2: 33.0%, n = 37/112). Over the study period, the percentage of participants reporting no casual sex partner increased significantly from 3.9% (n = 6/154) at baseline to 22.1% (n = 34/154) at FU1 and FU2 (p < 0.001). Moreover, the percentage of individuals reporting sometimes using condoms and always using PrEP during anal sex with a casual partner decreased significantly from baseline (38.5%, n = 55/143) to FU2 (33.0%, n = 37/112) (p < 0.001). Furthermore, the percentage of participants always using condoms and PrEP decreased significantly from baseline (7.7%, n = 11/143) to FU2 (2.7%, n = 3/112) (p = 0.028).

During the study, 162 (93.6%) participants indicated having had anal sex with anonymous partners in one or more questionnaires. At baseline, 48.0% (n = 61/127) of participants who reported anal sex with anonymous partners indicated never using condoms and always using PrEP compared to 43.0% (n = 58/135) at FU2; 37.0% (n = 47/127) to 43.7% (n = 59/135) sometimes using condoms and always using PrEP, respectively, and 12.6% (n = 16/127) to 8.1% (n = 11/135) always using condoms and PrEP, respectively.

Over the study period, the percentage of participants protected neither by PrEP nor by condoms during anal sex ranged between 29.0% (n = 18/62) and 33.3% (n = 23/69) with steady partners, between 0% (n = 0/114) and 2.7% (n = 3/112) with casual sex partners, and between 0% (n = 0/127) and 0.7% (n = 1/135) with anonymous sex partners.

## Discussion

This study among PrEP users, who were mainly MSM, in Belgium shows that in practice, distinct categories of PrEP use exist, going beyond the daily and on-demand distinction. We found associations between PrEP use categories and type and number of sexual partners. PrEP users were likely to remain in the same PrEP use category, though switches between these categories were also observed. Sexual acts not protected by condoms nor PrEP with casual or anonymous sex partners were rarely reported, but was more frequent with steady partners. Overall, condom use was relatively low and almost one-third of participants reported being diagnosed with any STI 6-months prior to the baseline questionnaire. These findings demonstrate that PrEP users have different patterns of PrEP and condom use and that these are associated with sexual behaviours with different partners.

To our knowledge, this is one of the first studies investigating patterns of PrEP use over time. A group-based trajectory modelling study among MSM and transgender women in the Netherlands and Belgium between 2015 and 2020 identified four trajectories based on the reported number of PrEP pills taken per week, ranging from low frequency use to daily use [23]. Similar to our findings, high proportions of users reported using PrEP in a consistent manner over time. Nevertheless, we also observed transitions to other categories of use, mainly adjacent categories, which is in line with previous studies on PrEP regimen use [5, 6, 24]. Our study reaffirms the association between sexual behaviour and how PrEP is used, with daily users reporting more casual and anonymous sex partners and a higher frequency of anal sex with these partners compared to long and short period users [8, 24–26]. This finding suggests that PrEP users know how to adapt their PrEP use to meet their needs.

This study provides additional evidence that most PrEP users report never or occasionally using condoms when taking PrEP during anal sex with casual or anonymous partners and thus are at risk for acquiring STIs other than HIV [8, 9, 27, 28]. A minority of participants reported always using condoms and PrEP concurrently with casual or anonymous partners. As shown in a systematic review, PrEP users experience PrEP as a facilitator to physical closeness and sexual pleasure and thus choose not to use condoms [29]. Mixed-methods and qualitative studies among MSM demonstrate that the decision to use condoms is driven by a trade-off between additional protection against HIV/STIs and increased sexual pleasure [11, 27, 30]. Although PrEP use in isolation often seems to be the preferred HIV prevention option among PrEP users, condoms remain one of the most effective and widely available STI prevention tools [31]. With increasing incidence of STIs among MSM, control and prevention of STIs are a public health priority [28]. Therefore, the promotion of condoms among MSM who use PrEP remains important to reduce the rate of STIs. These findings on PrEP and condom use could help guide who to target for tailored PrEP care. For example, a group that could be targeted are those PrEP users who never use condoms, but always use PrEP with anonymous sex partners. For these individuals, condom promotion and offering STI testing services can be of added value to their PrEP care. Further investments and research are also needed to explore and develop STI prevention strategies that allow for physical closeness and sexual pleasure.

The low percentage of participants reporting never using condoms and PrEP for anal sex acts with casual and anonymous partners highlights that only a minority of PrEP users in this study are not effectively protected against HIV acquisition during anal sex with these partners. A mixed-methods study on condom use among PrEP users in the Netherlands similarly showed that 1.4% of sex acts with known casual partners and 1.2% of sex acts with unknown casual partners were without use of condoms or PrEP [27]. By contrast, a French cohort study found that about 13% of on-demand PrEP users reported no PrEP or condom use at their most recent sexual act in the preceding three months, whereas this was 3% among participants using daily PrEP [25]. The latter study did not specify the type of sexual acts and type of partners. If we are to maximise the potential of PrEP, we recommend enhanced approaches to differentiate these PrEP users likely to have anal condomless sex without PrEP with casual or anonymous partners, and provide tailored follow-up such as PrEP adherence support.

About one in three participants in our study reported never using condoms or PrEP during anal sex with a steady partner. These findings are in line with a Dutch study on condom use among PrEP users [27], which showed that 25% of sex acts with steady partners were without condoms or PrEP. The open-label extension phase of the ANRS IPERGAY trial found similar patterns, with 85% of sexual acts with main sexual partners without condoms or PrEP [32]. Our findings confirm that PrEP users take into account the type of partner during HIV prevention decision-making [11, 27, 32]. Applying such HIV risk management strategy is not without risks. Modelling studies estimated that between 32% and 68% of HIV infections among MSM stem from sexual intercourses within main relationships [33, 34], in particular because of non-exclusive sex [35], and the high prevalence of HIV among MSM [2]. Hence, it is essential to consider the relationship context to tailor prevention recommendations as HIV serostatus and PrEP status of the dyad and their sexual agreement influence their HIV acquisition risk [35, 36]. Couples-based HIV prevention interventions, such as couples HIV testing and counselling and establishing and adhering to sexual agreements, have proven to be effective in reducing HIV risk [37–39] and should be integrated in PrEP programmes.

A first limitation of this study is that, inherent to online surveys, we cannot exclude self-selection bias. Individuals associated with LGBTQI or sexual health organisations were more likely to be recruited due to the chosen recruitment strategies (e.g. social media platforms of MSM community). Therefore, it is unlikely that our study population is a random and representative sample of all PrEP users in Belgium. Second, many participants did not complete all three study rounds, and were thus excluded from the longitudinal analyses. This likely created a selection bias, particularly as, participants completing all study rounds were older and reported more consistent PrEP use during anal sex with casual or anonymous partners at baseline compared to those who did not complete all rounds. This could have led to an overestimation of the percentage of sex acts covered by PrEP and condoms. Third, due to the limited sample size, we were not able to examine associations between sociodemographic and sexual behaviour characteristics, and patterns of PrEP use over time. Fourth, the 6-monthly intervals did not allow us to verify effective PrEP use (i.e. the appropriate use of PrEP during periods of HIV risk to achieve high levels of protection against HIV acquisition), which requires more detailed data such as timing of sexual acts and PrEP intake. Fifth, we lacked information on participants’ knowledge of the HIV status and use of antiretroviral therapy of their sexual partners, information which in all likelihood would have influenced their PrEP and condom use. Finally, this study started during the COVID-19 pandemic, and its related restrictions. These restrictions could have impacted sexual behaviour[26], and therefore PrEP and condom use, which might have affected our results and their generalizability to other PrEP users.

Nevertheless, our study recruited 8.2% (326/3986) of registered PrEP users in Belgium in 2020, irrespective of the clinic where they were prescribed PrEP [40]. The percentage of men (99.1% vs. 99.2%) and daily or almost daily users (43.6% vs. 38.7%) in our sample were comparable to percentages reported in the national PrEP users’ population in 2020 [40]. Giving the paucity of longitudinal studies with different oral PrEP regimen options outside the scope of clinical trials, our study provides valuable insights into the variability of PrEP and condom use by sexual partner type over time. Such insights are important to better understand how PrEP care can be tailored to such patterns.

## Conclusions

We identified five distinct patterns in PrEP use ranging from daily use, to episodes of seven or fewer consecutive days of use, to no PrEP use. Most PrEP users remained in the same PrEP use category throughout the study and PrEP use was associated with sexual behaviours. We advocate for moving away from the regimen dichotomy of daily and on-demand use, and to provide tailored care for different types of PrEP users to enhance the effectiveness of PrEP programmes and to support adherence to PrEP based on users’ needs and preferences. While overall condom use was relatively low, condomless sex without PrEP with casual or anonymous partners were rare, but more frequent during sex with steady partners. This suggests room for targeted counselling, such as couples-based HIV prevention interventions, considering the potentially high risk of acquiring HIV in a steady relationship. Further research is needed to investigate how PrEP care could be tailored according to different PrEP and condom use patterns.

## Electronic supplementary material

Below is the link to the electronic supplementary material.

Supplementary Material 1

## Data Availability

The datasets generated during and/or analysed during the current study are not publicly available, but are available upon reasonable request to the corresponding author and if approved by the Institutional Review Board of the Institute of Tropical Medicine (Antwerp).

## List of Abbreviations

IQR: Interquartile range
IRB: Institutional review board
FU: Follow-up questionnaire
MSM: Men who have sex with men
PrEP: Pre-exposure prophylaxis
SD: Standard deviation
STI: Sexually transmitted infections
