# Supplementary material for: Patterns of PrEP and condom use among PrEP users in Belgium: a web-based longitudinal study
Source: BMC Public Health. 2023 May 26;23:970. doi: 10.1186/s12889-023-15786-6 (PMC10214320; doi:10.1186/s12889-023-15786-6)
Supplement: Supplementary file 1 — Supplementary Material 1 [file 12889_2023_15786_MOESM1_ESM.docx]

**Appendix to**

**Patterns of PrEP and condom use among PrEP users in Belgium: a web-based longitudinal study**

*Authors*

Anke Rotsaert^1§^, Tom Smekens^1^, Bea Vuylsteke^1^, Maarten Schim van der Loeff^2,3,4,5^, Bernadette Hensen^1^, Christiana Nöstlinger^1^, Edwin Wouters^6^, Jef Vanhamel^1^, Gert Scheerder^1^, Thijs Reyniers^1^

*Affiliation*

1. Department of Public Health, Institute of Tropical Medicine, Antwerp, Belgium
2. Department of Infectious Diseases, Research and Prevention, Public Health Service of Amsterdam, Amsterdam, The Netherlands
3. Amsterdam UMC location University of Amsterdam, Department of Internal Medicine, Meibergdreef 9, Amsterdam, The Netherlands
4. Amsterdam Institute for Infection and Immunity (AII), Amsterdam, the Netherlands
5. Amsterdam Public Health Research Institute (APH), Amsterdam, the Netherlands
6. Department of Sociology, University of Antwerp, Antwerp, Belgium

§ *Corresponding Author*: Anke Rotsaert

Department of Public Health

Institute of Tropical Medicine

Nationalestraat 155

Antwerp, 2000, Belgium

+32497415860

arotsaert@itg.be

**Additional file 1. Sexual health scale**^1^

To what extent do you disagree or agree with the following statements?

1. I am happy with my sex life.
2. Strongly disagree – (2) disagree – (3) neutral or uncertain – (4) agree – (5) strongly agree
3. The sex I have is always as safe as I want it to be.
4. Strongly disagree – (2) disagree – (3) neutral or uncertain – (4) agree – (5) strongly agree
5. I find it easy to say no to sex I don’t want.
6. Strongly disagree – (2) disagree – (3) neutral or uncertain – (4) agree – (5) strongly agree
7. I am sexually as confident as I would like to be.
8. Strongly disagree – (2) disagree – (3) neutral or uncertain – (4) agree – (5) strongly agree

Scoring involved summing the items and taking the mean response (sum of items/4).

**Additional file 2. Sexual sensation seeking scale**^2^

A number of statements that some people have used to describe themselves are given below. Read each statement and then select the number to show how well you believe the statement describes you.

1. I like wild ‘unhibited’ sexual encounters.

(1) Not at all like me – (2) slightly like me – (3) mainly like me – (4) very much like me

1. The physical sensations are the most important thing about having sex.

(1) Not at all like me – (2) slightly like me – (3) mainly like me – (4) very much like me

1. My sexual partners probably think that I take many risks in general.

(1) Not at all like me – (2) slightly like me – (3) mainly like me – (4) very much like me

1. When it comes to sex, physical attraction is more important to me than how well I know the person.

(1) Not at all like me – (2) slightly like me – (3) mainly like me – (4) very much like me

1. I enjoy the company of sensual people.

(1) Not at all like me – (2) slightly like me – (3) mainly like me – (4) very much like me

1. I enjoy watching porn movies.

(1) Not at all like me – (2) slightly like me – (3) mainly like me – (4) very much like me

1. I am interested in trying out new sexual experiences.

(1) Not at all like me – (2) slightly like me – (3) mainly like me – (4) very much like me

1. I feel like exploring my sexuality.

(1) Not at all like me – (2) slightly like me – (3) mainly like me – (4) very much like me

1. I like to have new and exciting sexual experiences and sensations.

(1) Not at all like me – (2) slightly like me – (3) mainly like me – (4) very much like me

1. I enjoy the sensations of intercourse without a condom.

(1) Not at all like me – (2) slightly like me – (3) mainly like me – (4) very much like me

Scoring involved summing the items and taking the mean response (sum of items/10).

**References**

1. Reyniers T, Nöstlinger C, Vuylsteke B, De Baetselier I, Wouters K, Laga M. The Impact of PrEP on the Sex Lives of MSM at High Risk for HIV Infection: Results of a Belgian Cohort. *AIDS Behav*. 2021;25(2):532-541. doi:10.1007/S10461-020-03010-0

2. Kalichman SC. Sexual sensation seeking scale. *Handb Sex Meas*. September 2013:564-565. doi:10.4324/9781315881089-74/SEXUAL-SENSATION-SEEKING-SCALE-KALICHMAN
